# Supplementary material for: A content analysis of 32 years of Shark Week documentaries
Source: PLoS One. 2022 Nov 3;17(11):e0256842. doi: 10.1371/journal.pone.0256842 (PMC9632781; doi:10.1371/journal.pone.0256842)
Supplement: S1 Table — Blue column indicates whether the episode was included in the content analysis and the initials of the coder that analyzed the episode. Yellow columns indicate titles deemed negative using the Affective Norms for English Words (ANEW), using context (phrases instead of individual words), and with the ANEW and context combined. (PDF) [file pone.0256842.s001.pdf]

SUPPLEMENT 1: SHARK WEEK EPISODES BY YEAR

Year	Title	Included in content analysis	Original coder	Included in redundancy analysis	Deemed negative - ANEW	Deemed negative - Context	Deemed negative - Combined
**1988**	Caged in Fear				X		X
**1988**	Sharks - Predators or Prey						
**1988**	The Shark Takes a Siesta						
**1988**	Sharks of a Different Color						
**1989**	Shark: Maneater or Myth?				X		X
**1989**	Shark Hunters of Achill Island						
**1989**	Sharks of San Francisco						
**1990**	Shark! The World of Sharks and Barracuda						
**1990**	Sharks: Hunters of the Oceans						
**1991**	Shark Shooters						
**1991**	Sea Lovers						
**1991**	Great Shark Hunt	X	JS				
**1992**	The Man Who Loves Sharks						
**1992**	Sharks of Polynesia						
**1992**	Great White Encounters						
**1992**	Great White! - 1						
**1992**	Great White! - 2						
**1993**	African Shark Safari						
**1993**	Sharks on the Brink of Extinction						
**1993**	Teeth of Death				X		X
**1993**	Assignment Adventure						
**1993**	Sharks of Pirate Island						
**1994**	Great White Down Under						
**1994**	To Be With Sharks						
**1994**	Shark Attack Files	X	JS		X		X
**1994**	Shark Doctors						
**1995**	Sharks of the						

	Red Triangle						
**1995**	Rendezvous at Ningaloo						
**1995**	In Search of the Golden Hammerhead						
**1995**	Shark Attack Files	*repeat*					
**1996**	The Ultimate Guide: Sharks	X	BLM	X			
**1996**	Danger Beach				X		X
**1996**	Tales of the Tiger Shark	X	JS				
**1997**	Shark Bites: A Decade of Shark Week				X		X
**1997**	Swift, Smart and Deadly				X		X
**1997**	Sharks of the Wild Coast						
**1997**	In South African Waters						
**1997**	Shark Attack Files II				X		X
**1998**	Prehistoric Sharks	X	LBW				
**1998**	Zambezi Shark						
**1998**	Hammerheads: Nomads of the Sea						
**1998**	Great White: In Search of the Giants						
**1998**	Sharks of the Atlantic	X	BLM	X			
**1999**	Live From a Shark Cage						
**1999**	The Secret Life of Sharks						
**1999**	Big Tooth: Dead or Alive				X		X
**1999**	Sharks of the Deep Blue						
**1999**	Sharks in a Desert Sea	X	LBW				
**2000**	Sharks 3-D						
**2000**	Air Jaws: Sharks of South Africa	X	JS				
**2000**	Giant Sharks						
**2000**	Jurassic Shark	X	JS	X			
**2000**	Future Shark	X	LBW				
**2001**	Air Jaws: Sharks of South Africa	*repeat*					

**2001**	Paradise for Predators: Sharks of Palau						
**2001**	Shark Attack Files III				X		X
**2001**	10 Deadliest Sharks	X	JS		X		X
**2001**	Future Shark	*repeat*					
**2002**	Air Jaws II: Even Higher	X	JS				
**2002**	Great White Attack: A True Story				X		X
**2002**	Shark Attack Rescuers	X	LBW		X		X
**2002**	Shark Attack Files IV: Summer of the Shark				X		X
**2003**	Anatomy of a Shark Bite	X	LBW				
**2003**	Sharks Under Glass	X	JS				
**2003**	Monster Garage: Shark Boat						
**2003**	Diary of a Shark Man	X	JS				
**2003**	Robo Shark	X	JS				
**2003**	Shark Roulette	X	JS	X		X	X
**2003**	Jaws of the Pacific	X	LBW			X	X
**2004**	Primal Scream	X	LBW		X		X
**2004**	Tiger Shark Attack: Beyond Fear	X	LBW		X		X
**2004**	Monster House: Shark House						
**2004**	Great White Shark: Uncaged	X	JS				
**2004**	Dive to Shark Volcano	X	LBW				
**2004**	Bull Shark: World's Deadliest Shark	X	LBW		X		X
**2004**	Sharks: Size Matters	X	JS				
**2005**	MythBusters: Jaws Special	X	BLM				
**2005**	Sharkbite! Surviving Great Whites	X	LBW		X		X
**2005**	American Shark	X	LBW				
**2005**	Shark Hunter - Chasing the	X	LBW				

	Great White						
**2006**	Dirty Jobs: Jobs That Bite						
**2006**	Shark Attack Survivors	X	LBW		X		X
**2006**	Perfect Shark	X	LBW				
**2006**	Sharks: Are They Hunting Us?					X	X
**2006**	Shark Rebellion	X	LBW				
**2006**	Dirty Jobs: Jobs that Bite Harder						
**2006**	Science of Shark Sex						
**2007**	Ocean of Fear: The Worst Shark Attack Ever	X	BLM		X		X
**2007**	Deadly Stripes: Tiger Sharks	X	JS		X		X
**2007**	Top 5 Eaten Alive	X	BLM	X	X		X
**2007**	Shark Feeding Frenzy	X	BLM		X		X
**2007**	Perfect Predators	X	BLM	X			
**2007**	Shark Tribe	X	BLM				
**2007**	Sharks: A Family Affair		BLM				
**2007**	Sharkman	X	BLM	X			
**2008**	MythBusters: Shark Special 2	X	JS				
**2008**	Surviving Sharks	X	JS	X		X	X
**2008**	Day of the Shark	X	JS				
**2008**	Dirty Jobs: Greenland Shark Quest	X	JS				
**2008**	How Not to Become Shark Bait	X	JS	X		X	X
**2008**	Mysteries of the Shark Coast	X	JS				
**2009**	Blood in the Water	X	JS		X		X
**2009**	Deadly Waters	X	JS		X		X
**2009**	Day of the Shark 2	X	JS				
**2009**	Sharkbite Summer	X	JS		X		X
**2009**	Great White Appetite	X	JS	X			
**2009**	Shark After Dark	X	JS				

**2009**	Man vs. Fish: Mako Shark						
**2009**	Shark Attack: A Boy's Fight to Survive				X		X
**2010**	Ultimate Air Jaws	X	JS				
**2010**	Into the Shark Bite	X	JS		X		X
**2010**	Man vs. Fish: Tiger Shark						
**2010**	Day of the Shark 3	X	JS				
**2010**	Shark Bites: Adventures in Shark Week	X	JS		X		X
**2010**	Shark Bite Beach	X	JS		X		X
**2010**	Shark Attack Survival Guide	X	JS		X		X
**2011**	Great White Invasion	X	JS		X		X
**2011**	Jaws Comes Home	X	JS				
**2011**	Rogue Sharks	X	JS			X	X
**2011**	Summer of the Shark	X	JS				
**2011**	Killer Sharks: The Attacks of Black December	X	JS		X		X
**2011**	How Sharks Hunt	X	JS				
**2011**	Shark City	X	JS				
**2011**	When Fish Attack 3				X		X
**2012**	Air Jaws: Apocalypse	X	JS			X	X
**2012**	Shark Week's Impossible Shots	X	JS				
**2012**	Sharkzilla	X	JS			X	X
**2012**	MythBusters' Jawsome Shark Special	X	JS				
**2012**	How Jaws Changed the World	X	JS				
**2012**	Adrift: 47 Days With Sharks	X	JS				
**2012**	Shark Fight	X	JS			X	X
**2012**	World's Scariest Animal Attacks				X		X
**2012**	Great White Highway:	X	JS	X			

	Where the White Sharks Go						
**2012**	Shark Week's 25 Best Bites	X	JS		X		X
**2013**	Air Jaws: Countdown to Shark Week 2013						
**2013**	Megalodon: The Monster Shark Lives	X	JS			X	X
**2013**	Return of Jaws	X	JS			X	X
**2013**	Voodoo Sharks	X	JS	X			
**2013**	I Escaped Jaws	X	JS	X		X	X
**2013**	Spawn of Jaws	X	JS				
**2013**	Top 10 Sharkdown	X	JS				
**2013**	Great White Serial Killer	X	JS		X		X
**2013**	Sharkpocalypse	X	JS			X	X
**2013**	Alien Monster Shark: Alien Sharks of the Deep	X	JS				
**2013**	Great White Gauntlet	X	JS				
**2014**	Air Jaws: Fins of Fury	X	JS	X	X		X
**2014**	Shark of Darkness: Wrath of Submarine	X	JS		X		X
**2014**	Jaws Strikes Back	X	JS				
**2014**	Monster Hammerhead	X	JS	X			
**2014**	Alien Sharks: Return to the Abyss	X	JS				
**2014**	Lair of the Mega Shark	X	JS				
**2014**	Zombie Sharks	X	JS			X	X
**2014**	Spawn of Jaws: The Birth	X	JS			X	X
**2014**	I Escaped Jaws 2	X	JS				
**2014**	Sharkageddon	X	JS				
**2014**	Megalodon: The Extended Cut						
**2014**	Megalodon: The New Evidence	X	JS				
**2014**	Great White	X	JS				

	Matrix						
**2014**	Sharksanity	X	JS				
**2015**	Shark Trek	X	BLM				
**2015**	Island of the Mega Shark	X	BLM				
**2015**	Monster Mako	X	BLM			X	X
**2015**	Return of the Great White Serial Killer	X	BLM		X		X
**2015**	Alien Sharks: Close Encounters	X	BLM				
**2015**	Bride of Jaws	X	BLM			X	X
**2015**	Tiburones: Sharks of Cuba	X	BLM				
**2015**	Super Predator	X	BLM			X	X
**2015**	Ninja Sharks	X	BLM				
**2015**	Shark Planet						
**2015**	Sharks of the Shadow land	X	BLM				
**2015**	Shark Clans						
**2015**	Sharksanity 2	X	BLM	X		X	X
**2015**	Shark Island	X	BLM				
**2015**	Shark Alley: Legend of Dynamite	X	BLM				
**2015**	Air Jaws: Walking with Great Whites						
**2015**	MythBusters vs. Jaws	X	BLM				
**2016**	Tiger Beach	X	BLM				
**2016**	Return of the Monster Mako	X	BLM	X		X	X
**2016**	Isle of Jaws	X	BLM			X	X
**2016**	Shallow Water Invasion	X	BLM		X		X
**2016**	Jaws of the Deep	X	BLM			X	X
**2016**	Sharks Among Us	X	BLM				
**2016**	Wrath of a Great White Serial Killer	X	BLM		X		X
**2016**	Air Jaws: Night Stalker	X	BLM			X	X
**2016**	Deadliest Sharks	X	BLM		X		X
**2016**	Sharks vs. Dolphins: Face Off	X	BLM				
**2016**	Nuclear Sharks	X	BLM				
**2016**	Jungle Shark	X	BLM				
**2016**	Shark Bait	X	BLM			X	X

**2016**	Blue Serengeti	X	BLM				
**2016**	Sharksanity 3	X	BLM			X	X
**2016**	The Killing Games	X	BLM		X		X
**2017**	Great White serial Killer Lives	X	BLM		X		X
**2017**	Phelps vs. Shark: Great Gold vs Great White	X	BLM				
**2017**	Shark-Croc Showdown	X	BLM				
**2017**	Great Hammerhead Invasion	X	BLM		X		X
**2017**	Shark Vortex	X	BLM			X	X
**2017**	Return to the Isle of Jaws	X	BLM			X	X
**2017**	Alien Sharks: Stranger Fins	X	BLM				
**2017**	Sharks and the City: LA	X	BLM				
**2017**	Sharks and the City: NYC						
**2017**	The Lost Cage	X	BLM				
**2017**	Devil Sharks	X	BLM			X	X
**2017**	Shark Exile	X	BLM				
**2017**	Shark Storm	X	BLM	X		X	X
**2017**	African Shark Safari	X	BLM				
**2017**	Lair of the Sawfish	X	BLM			X	X
**2017**	Sharkmania	X	BLM			X	X
**2017**	Shark School with Michael Phelps	X	BLM				
**2018**	Shark Weeks 50 Best Bites	X	BLM				
**2018**	Ridiculous Cakes: 30th Anniversary of Shark Week						
**2018**	Bear vs. Shark	X	BLM				
**2018**	Shaq Does Shark Week	X	BLM				
**2018**	Ronda Rousey Uncaged	X	BLM				
**2018**	Monster Tag	X	BLM			X	X
**2018**	Great White Abyss	X	BLM			X	X
**2018**	Cuba's Secret Shark Lair	X	BLM			X	X
**2018**	Guy Fieri's	X	BLM		X		X

	Feeding Frenzy						
**2018**	Laws of Jaws	X	BLM			X	X
**2018**	Air Jaws: The Hunted	X	BLM			X	X
**2018**	Air Jaws: Back from the Dead	X	BLM			X	X
**2018**	Shark Tank Meets Shark Week	X	BLM				
**2018**	Sharkcam Stakeout	X	BLM	X			
**2018**	Sharkcam Strikes Back	X	BLM				
**2018**	Sharkwrecked	X	BLM			X	X
**2018**	Tiger Shark Invasion	X	BLM	X	X		X
**2018**	Cash Cab Shark Week Edition						
**2018**	Megalodon: Fact Vs. Fiction	X	BLM				
**2018**	Bloodline: Spawn of Jaws	X	BLM			X	X
**2018**	Great White Shark Babies	X	BLM				
**2018**	Return of the Mega Shark	X	BLM				
**2018**	Sharks Gone Wild	X	BLM			X	X
**2018**	Naked and Afraid of Sharks						
**2019**	Expedition Unknown: Megalodon	X	BLM				
**2019**	Shark Trip: Eat. Prey. Chum						
**2019**	Sharks of the Badlands	X	BLM				
**2019**	Legend of Deep Blue	X	JS				
**2019**	Sharks of Headstone Hell	X	JS				
**2019**	Sharkwrecked: Crash Landing	X	BLM			X	X
**2019**	Laws of Jaws: Dangerous Waters	X	JS		X		X
**2019**	Air Jaws Strikes Back	X	JS				
**2019**	Extinct or Alive: The Lost Shark	X	BLM				
**2019**	Capsized: Blood in the Water	X	JS	X	X		X
**2019**	Return to Shark	X	JS				

	Island						
**2019**	Great White Kill Zone: Guadalupe	X	JS		X		X
**2019**	Monster Mako: Perfect Predator	X	BLM			X	X
**2019**	Isle of Jaws: Blood Brothers	X	JS		X		X
**2019**	Andrew Mayne: Ghost Diver	X	JS				
**2019**	I was Prey: Shark Week	X	JS		X		X
**2019**	Sharks Gone Wild 2	X	JS			X	X
**2019**	Shark Week Immersion	X	JS				
**2020**	Air Jaws: Ultimate Breach Off	X	DSS				
**2020**	Tyson vs. Jaws: Rumble on the Reef	X	DSS				
**2020**	Shark Lockdown	X	DSS				
**2020**	Abandoned waters	X	DSS			X	X
**2020**	Shaq Attack	X	DSS		X		X
**2020**	Jaws Awakens	X	DSS			X	X
**2020**	Extinct or Alive: Land of the Lost Sharks	X	DSS				
**2020**	Will Smith: Off the Deep End	X	DSS				
**2020**	Great White Serial Killer: Extinction	X	DSS		X		X
**2020**	Monster Under the Bridge	X	DSS			X	X
**2020**	Adam Devine's Secret Shark Lair	X	DSS			X	X
**2020**	Great White Double Trouble	X	DSS				
**2020**	Air Jaws 2020						
**2020**	Snoop Dogg's Sharkadelic Summer						
**2020**	Mako Nation	X	DSS				
**2020**	Alien Sharks: First Contact	X	DSS				
**2020**	Lair of the Great White	X	DSS			X	X
**2020**	Tiger Shark King	X	DSS				

**2020**	I Was Prey 2	X	DSS			X	X
**2020**	Sharks of ghost island	X	DSS				
**2020**	Wicked Sharks	X	DSS		X		X
**2020**	Sharks Gone Wild 3					X	X
**2020**	I Was Prey: Terror of the Deep	X	DSS		X		X
**2020**	Naked and Afraid of Sharks 2						
