# Supplementary material for: A content analysis of 32 years of Shark Week documentaries
Source: PLoS One. 2022 Nov 3;17(11):e0256842. doi: 10.1371/journal.pone.0256842 (PMC9632781; doi:10.1371/journal.pone.0256842)
Supplement: S2 Table — (PDF) [file pone.0256842.s002.pdf]

SUPPLEMENT 3: SHARK SPECIES FEATURED ON SHARK WEEK

Extant shark species with appearances in more than 5 episodes

Shark species	N	%
Great white	146	18.43%
Tiger shark	97	12.25%
Bull	76	9.60%
Hammerhead	67	8.46%
Lemon	53	6.69%
Caribbean reef	50	6.31%
Nurse	41	5.18%
Mako	37	4.67%
Blacktip	35	4.42%
Blue shark	21	2.65%
Whale	20	2.53%
Oceanic whitetip	20	2.53%
Sand tiger	19	2.40%
Whitetip reef shark	18	2.27%
Grey reef	14	1.77%
Silky shark	12	1.52%
Galapagos	11	1.39%
Megamouth	9	1.14%
Goblin	8	1.01%
Cookiecutter	8	1.01%
Thresher	7	0.88%
Spinner shark	7	0.88%
Silvertip	7	0.88%
Sandbar	7	0.88%
Bronze whaler	7	0.88%
Gulper	6	0.76%
Greenland	6	0.76%
Bonnethead	5	0.63%
Blacktip reef	5	0.63%
Angel shark	5	0.63%
Frilled	5	0.60%

All chondrichthyan species (extant and extinct) featured on Shark Week

Species	N
Angel shark	5
Antarctic lamni (extinct)	1
Atlantic stingray	1
Austin's guitarshark	1
Bat ray	1
Basking shark	4
Big eyed ragged tooth	1
Birdbeak dogfish	1
Blacknose shark	2
Blacktip reef shark	5
Blacktip shark	35
Blue shark	21
Bonnethead	5
Bronze whaler	7
Brownbanded bamboo shark	2
Bull shark	76
Bullhead shark	1
Caribbean reef shark	50
Carpet shark	1
Chained dogfish	1
Chimera	1
Clearnose skate	2
Common sawshark	1
Cook shark	1
Cookiecutter shark	8
Dusky shark	3
Eagle ray	1
Edestus (extinct)	1
Elephant fish	1
Epaulette shark	1
Finetooth shark	1
Frilled shark	5
Galapagos shark	11
Ghost shark	3
Goblin shark	8
Great white shark	146
Greenland shark	6

Grey reef shark	14
Guitarfish	2
Gulper shark	6
Hammerhead	67
Hardnosed shark	1
Helicoprion (extinct)	2
Horn shark	2
Iniopterygiians (extinct)	1
Kitefin	3
Lantern shark	1
Lemon shark	53
Leopard shark	2
Longnose dogfish	1
Mako	37
Mandarin dogfish	1
Manta	4
Marble ray	1
Megaldon (extinct)	12
Megamouth	9
Mexican horn shark	1
Milk shark	1
Night shark	1
Nurse shark	41
Oceanic whitetip shark	20
Orthcanthus (extinct)	1
Pacific sleeper	3
Pigeye shark	2
Pocket shark	1
Pondicherry shark	1
Porbeagle	2
Puffadder shyshark	3
Pyjama shark	2
Roughskin dogfish	1
Salmon shark	3
Sand tiger	19
Sandbar shark	7
Sawfish	4
Sevengill	4
Sharpnose	1

Shorttail nurse	1
Silky shark	12
Silvertip shark	7
Sixgill	4
Sleeper shark	1
Smalltooth sawfish	1
Smooth-hound	2
Southern stingray	4
Spinner shark	7
Spiny dogfish	1
Spotted ragged tooth	1
Stethacanthus (extinct)	3
Swell shark	3
Tawny nurse shark	1
Thresher	7
Tiger shark	97
Torpedo ray	1
Unicorn shark (extinct)	1
Viper shark	1
Whale shark	20
Whaler	1
Whitetip reef shark	18
Whitetip weasel shark	1
Wobbegong	3
Zebra shark	5
