# Supplementary material for: A content analysis of 32 years of Shark Week documentaries
Source: PLoS One. 2022 Nov 3;17(11):e0256842. doi: 10.1371/journal.pone.0256842 (PMC9632781; doi:10.1371/journal.pone.0256842)
Supplement: S1 File — (PDF) [file pone.0256842.s003.pdf]

SUPPLEMENT 2: CODING GUIDELINES FOR CONTENT ANALYSIS OF SHARK WEEK EPISODES

General information:
Each episode was viewed and coded by one of four evaluators (BM, JS, DS, LBW) using a Google form. To minimize subjectivity, author LBW did not code the episode that she appeared in. If the coder was unsure about a particular category, they noted that in the form for further review by DS, who acted as arbitrator and performed analyses on the content data.

Variables:

A) Title of Documentary: Recorded exactly from the title screen.

B) Year of documentary: Year the documentary aired.

C) What production company made the show? Recorded from end credits when present.

D) Location of filming: Where the documentary took place, as stated in the documentary or appearing in text on screen. Specifically, we were looking for localities that were featured beyond an interview in someone's workplace. In some cases, documentaries took place in more than one location; all locations were recorded for this variable. Locations ranged from vague to specific.

Examples: The Pacific Ocean
South Africa
Volusia County (United States)
Bondi Beach (Australia)

E) Chondrichthyan species: Chondrichthyans (sharks, skates, rays, chimeras) that were featured and mentioned by name, either as the focus of an entire show or of a short scene within the show. Species were usually referred to by common name, although occasionally scientific names were used. In some cases, more than one species was featured; all species featured were recorded for this variable.

Examples: Great white shark
Hammerhead
Ratfish

F) Experts and hosts: Names, credentials, and affiliations of people interviewed in the documentary. Often, this was written as text across the bottom of the screen. This information was recorded as it was presented in the documentary (i.e., if someone was presented as a marine biologist they were recorded as a marine biologist, we did not independently confirm people's credentials, and we note that Shark Week features some credentialed experts who are viewed as unreliable fringe figures in the wider scientific community). In some cases, more than one person was featured; all people featured were recorded for this variable. If no people were featured, the variable was left blank.

Examples: Dr. Toby Daly-Engel, evolutionary biologist (categorized as "scientist")
Alison Towner, shark biologist (categorized as "scientist")
Jeff Kerr, Discovery producer (categorized as "non-scientist")
Andy Casagrande, shark expert (categorized as "non-scientist")

G) <u>General type of documentary</u>:

A) Natural history: These shows focused entirely on the wild, natural behavior of a species (or group of species) of sharks, and include no research methods other than observation and photography. A scientist may or may not have been interviewed, a question may or may not been asked, the question may or may not have been answered.

B) Research focus: These shows focused on a scientist or team(s) of scientists with a specific research question as they attempt to answer that question, through field and/or laboratory research. The question may or may not have been answered. If the people answering the question were not trained credentialed scientists, this was a Category E program not a Category B show.

C) Shark bites: These shows focused on stories of sharks biting people, sometimes with re-enactments, sometimes with interviews of witnesses or victims or victim's families. Experts in shark bites are sometimes but not always consulted.

D) Clip show: These shows recycled existing footage (either from past Shark Week shows or from other sources), often in a "countdown" or "best of" type format.

E) Diving with sharks: These shows featured a host, who is not a trained credentialed expert but claims to be an expert, who has a question about shark biology or behavior. They do not ask a trained credentialed expert, but instead seek out investigating the question on their own, such as by cage diving with great white sharks and claiming to be observing that behavior. These are distinct form Category B shows, which may sometimes involve shark diving but are not focused on it. These are also distinct from Category A shows because they're framed around a person with a question who goes diving, rather than just describing an existing well-known behavior.

F) Mythical/monster/legendary shark: These shows are about a search for a specific individual shark often associated with some local legend and named.

G) Other: Anything that doesn't neatly fit into one of the above categories classes was recorded as "other" with a brief but detailed description of what they were about.

Examples:

The 2011 documentary "Rogue Sharks" focused on dramatic recreations of sharks biting people including interviewing victims and witnesses, and was classified as "shark bites."

The 2015 show "Alien Sharks: Close Encounters focused on scientists studying unusual and rarely seen species, and was classified as "research".

2020's "Mike Tyson: Rumble on the Reef" featured celebrity boxer Mike Tyson swimming with sharks for no discernible educational purpose, and was classified as 'Swimming with sharks."

The Megalodon series, which falsely claimed that *Otodus megalodon* is still swimming in the ocean, was classified as mythical/legendary sharks.

H) Research featured: Any research methods used in the documentary were recorded, including SCUBA diving videography. In some cases, multiple research methods were used; all research methods used were recorded for this variable.

Examples:

Underwater photography (though we note that filming a shark is not necessarily studying a shark)

Satellite telemetry tagging

I) Purpose/goal: The stated purpose of goal of the documentary often communicated within the first few minutes of the show. This information was recorded exactly as it was presented in the documentary.

Examples:

For example, 2014's "Spawn of Jaws: The Birth" focused on researcher Dr. Michael Domeier trying to track a pregnant great white to learn where great whites give birth. They did not find where great whites give birth, though they noted that they learned a lot about pregnant shark behavior in general.

2014's "Lair of the Megashark" focused on non-scientists Jeff Kerr and Andy Casagrande attempting to investigate supposed legends of a giant "mega shark" near New Zealand. They did not find it, as it likely does not exist.

J) Purpose/goal accomplished?: We assessed whether the stated goal of the program was accomplished, most often by whether the narrator or featured experts stated whether their goal was accomplished. See (I) above for examples.

K) Conservation: Any mention of topics related to shark conservation, including endangerment and extinction threat, were recorded as direct quotations.

Example: 2018's "AIr Jaws: The Hunted" included the line "Great whites are threatened by fisheries, global warming, loss of habitat, and poaching." (We note that great whites are notably the most protected from fisheries shark species in the world, and are not threatened by global warming or loss of habitat).

L) Shark fins: Any mentions of shark finning or humans eating shark fins were recorded as direct quotations.

Example: 2003's "Roboshark" noted "At least 100 million sharks die each year to be made into shark fin soup." (This figure is incorrect, see Shiffman et al. 2020).

M) Shark meat: Any mentions of humans eating shark meat were recorded as direct quotations.

Example: In 2000's "Future Shark," the narrator noted that "heavy hunting of whale sharks for their meat and fins has resulted in a worldwide decline in shark sightings."

N) Helping sharks: Any mentions of specific ways people can help sharks were recorded as direct quotations.

Example: 1991's "Great Shark Hunt" encouraged recreational anglers to release sharks they catch.

O) Negative portrayal of sharks: When sharks were portrayed as scary, dangerous, or bad in any way by either a narrator or interviewee, we recorded direct quotations.

Example: From 1996's "Tales of the Tiger Shark": "There is reason to fear the tiger shark, to hate the beast with vengeance, to mock, despite, and be repulsed by its hideous tooth griminess"

P) Positive portrayal of sharks: When sharks were portrayed as good or important for the ecosystem in any way by either a narrator or interviewee, we recorded direct quotations.

Example: From 2005's American Shark: "As scary as they look, these are gentle giants."

Q) Other portrayal of sharks: When sharks were portrayed as something other than good or bad, we recorded direct quotations.

R) Misconceptions: We were looking for two specific common misconceptions: "Bull sharks are the only sharks that can enter freshwater" and "Sharks can smell a drop of blood from a mile away." If some variation of either misconception was mentioned, we recorded direct quotations.
